# Mitigating the Risk of QTc Prolongation When Using Haloperidol for Acute Treatment of Cannabinoid Hyperemesis Syndrome in Adolescents and Young Adults

**DOI:** 10.3390/jcm14010163

**Published:** 2024-12-30

**Authors:** Sandra Merino, Lissette Tordera, Allison Jun, Sun Yang

**Affiliations:** 1 Department of Pharmacy, CHOC Children’s Hospital, Orange, CA 92868, USA; smoorhouse@choc.org (S.M.); lissette.tordera@choc.org (L.T.); ajun@choc.org (A.J.); 2 Department of Pharmacy Practice, Chapman University School of Pharmacy, Irvine, CA 92618, USA

**Keywords:** Cannabinoid Hyperemesis Syndrome (CHS), antiemetics, QTc prolongation, haloperidol, adolescents, young adults

## Abstract

**Background/Objectives**: Cannabinoid Hyperemesis Syndrome (CHS), associated with long-term cannabinoid use, has been increasingly observed in emergency room visits as more states in the U.S. have legislatively permitted medical and recreational marijuana use. The acute management of CHS primarily focuses on antiemetic treatment and supportive care. However, both the condition itself and the antiemetic drugs, such as haloperidol, may cause QTc prolongation. **Methods**: We reported two adolescent cases admitted to the emergency department for acute antiemesis management of CHS who received haloperidol treatment. A literature review was performed through October 2024 for previously published cases of QTc prolongation and/or Torsades de Pointes (TdP) in adolescents and young adults. **Results**: A 15-year-old female presented with hypokalemia and hypomagnesemia upon admission. She complained of chest pain and tachycardia, and the electrocardiogram (EKG) showed prolonged QTc (528 msec). The haloperidol infusion was discontinued. She recovered well post-discharge without complaints. A 17-year-old female had a borderline prolonged QT interval (476 msec). Her nausea and vomiting improved with a three-dose course of intravenous fosaprepitant before discharge. Our literature search identified five severe cases with life-threatening episodes of QTc prolongation and/or TdP in adolescents and young adults. **Conclusions**: Patients with CHS are at higher risk of QTc prolongation due to cannabis use, electrolyte imbalance, and antiemetic medications. We recommend vigilant EKG monitoring, particularly before initiating and throughout haloperidol treatment. If the patient presents with an increased risk of QTc prolongation, consider using topical capsaicin, lorazepam, aprepitant/fosaprepitant, and olanzapine as alternatives.

## 1. Introduction

Cannabis use has notably increased over the past decade. As of 24 April 2023, thirty-eight states, three territories, and the District of Columbia allow the medical use of cannabis products. Recreational use for adults older than 21 years is permitted in twenty-four U.S. states, two U.S. territories, and the District of Columbia [1]. With expanding availability, use is increasing in the pediatric population, especially among adolescents. The 2021 National Survey on Drug Use and Health study estimated that more than 2.6 million people aged 12 or older, particularly 869,000 adolescents (12 to 17 years old), initiated marijuana use in 2020 [2], which notably increased respectively to 3.7 million and 1.2 million in 2021 [3]. In 2022, 22.0 percent of individuals aged 12 or older, roughly 1 in 5, used marijuana in the past year, regardless of the mode of use [3]. Furthermore, the number of people ≥12 years old with a marijuana use disorder in the past year increased from 5.8% in 2021 to 6.7% in 2022, which is estimated at about 19.0 million people [3]. Such a notable increase may be attributed to the legalization of medical use and recreational use of marijuana across many states. Accompanied by the increase in prevalence, notable ethnic disparities remain consistent in cannabis use, with the highest rates observed among the American Indian or Alaska Native and Multiracial populations. In 2022, marijuana use among Multiracial, Black, Hispanic, White, and Asian populations increased markedly across all groups compared to 2021 [2,3].

Accumulating studies have linked cannabis use to significant adverse cardiac and cardiovascular events, such as heart failure, heart attack, and hypertension [4,5]. An earlier study reported that among young adult users of cannabis, the most prevalent arrhythmia was atrial fibrillation (42%) [6]. Mechanistic studies have also shown that cannabis use is associated with a prolonged QT interval due to the inhibition of the delayed rectifier potassium channel (hERG) [7,8]. In addition, chronic tetrahydrocannabinol (THC) use may result in increased parasympathetic and reduced sympathetic activity, resulting in bradycardia, which further predisposes to QTc prolongation [9,10]. A retrospective prevalence study analyzed admissions at over 4000 hospitals using International Classification of Diseases, Tenth Edition (ICD-10 CM) codes, which identified over 68,000 cannabis users aged 13–19 years [7]. QTc prolongation was the most common cardiac arrhythmia among teenage cannabis users (513.1 events per 100,000 patients). Sudden cardiac arrest, though rare, has been reported during cannabis use among adolescent and young adult cannabis users [10,11,12]. Of particular concern is the emergence of new synthetic forms of cannabis, which can be up to 10 times more potent. A method of wax vaping, otherwise known as “dabbing”, utilizes butane hash oil that has a THC concentration exceeding 50%, in contrast to the medicinal plant, which typically contains THC between 4% and 8%.

Cannabinoid Hyperemesis Syndrome (CHS), which may occur during frequent long-term cannabis use, has been on the rise in recent years [13]. CHS is characterized by pronounced cyclical nausea, intractable vomiting, and abdominal pain that often does not respond to traditional anti-nausea treatments. This persistent vomiting can put patients at risk for significant electrolyte imbalances that are recognized risk factors for QTc prolongation and Torsades de Pointes (TdP), such as hypokalemia [12]. In addition to hydration fluids and electrolyte correction, the primary acute management focuses on antiemesis [14,15]. A wide variety of antiemetics are often trialed in the emergency department (ED), with mixed results. Ondansetron is the most frequently used, although insufficient symptom relief is common [16]. Other commonly used traditional antiemetics, such as metoclopramide, promethazine, prochlorperazine, and antihistamines, have been tried with limited success [14]. Recent studies suggest that non-traditional antiemetics such as benzodiazepines and haloperidol may be most effective for the acute treatment of CHS, in addition to the topical application of capsaicin cream [14,15,17]. However, many antiemetics used for acute treatment carry a risk of QTc prolongation, with haloperidol being particularly concerning for this adverse effect [18]. Since both the condition itself and the treatments used may cause QTc prolongation and increase the risk of TdP, clinicians must remain vigilant in assessing and mitigating the risk of QTc prolongation while providing acute care for CHS.

Here, we present two cases of CHS treated at our emergency department with various risk factors for QTc prolongation, followed by a discussion of approaches we recommend to mitigate that risk and improve patient safety.

## 2. Case Presentation

Patient A, a 15-year-old female, was seen in the ED for reported abdominal pain, nausea, anxiety, and consistent panic attacks associated with a fear of eating. She had experienced an unintentional 30-pound weight loss over the previous 6 months, including 10-pound weight losses over the previous two weeks, denying any history of anorexia or bulimia. The patient had undergone exploratory laparoscopy ten days prior due to concern for esophageal perforation related to cyclic vomiting syndrome secondary to reported daily cannabis use. The family was working on finding a therapist for mental health. Her most recent marijuana use was four days prior to the ED visit. The patient’s referring primary care physician relayed prior improvement of agitation and anxiety with lorazepam. The urine drug screening (UDS) was positive for cannabinoids.

The patient was ordered aluminum hydroxide/magnesium hydroxide/simethicone oral solution, intravenous (IV) ondansetron 4 mg, and haloperidol 0.05 mg/kg IV, which she reported having received previously. ED prescribers ordered an EKG to rule out cardiac causes of her chest pain and tachycardia, which showed a QTc interval of 528 msec (ms). Fifteen minutes into the 45-min haloperidol infusion, after a review of the QTc interval, the haloperidol infusion was stopped. Labs were reviewed, indicating a serum potassium of 3 mmol/L and serum magnesium of 1.9 mg/dL. A change in priorities occurred during the ED visit as the time came closer to the patient’s pre-existing appointment with a psychiatrist for anxiety. The patient was given potassium 20 mEq PO and lorazepam 1 mg PO after the prescriber confirmed with the pharmacist that these are not QTc-prolonging medications. The pharmacist gave the patient’s mother a copy of the EKG with the QTc interval highlighted. The mother agreed to show the EKG to the psychiatrist, with the understanding that the psychiatrist would likely take it into consideration when choosing anxiety medications (e.g., selective serotonin reuptake inhibitors). The ED pharmacist reassured the patient that the psychiatrist would still have several safe medication options to treat her anxiety, including those that help immediately.

Patient B, a 17-year-old female visiting California, presented to our ED for intractable vomiting. The patient initially presented at an outside hospital where she received diphenhydramine and haloperidol (no dosing information available). The patient was then discharged after the vomiting was resolved. A day later, the patient was readmitted to the same outside hospital for the same complaints and received IV ondansetron and haloperidol. After discharge, the patient went home and had a cracker before her vomiting returned. The patient returned to an outside hospital and was ultimately transferred to our ED. She denied any blood in her emesis but endorsed a rusty brown color on admission. She had no prior diagnosis of cyclic vomiting or CHS but did periodically have a few days of vomiting with 1–2 episodes of nausea and emesis in the past, which usually resolved on their own. Additionally, she reported that hot baths helped. Such episodes occurred 6 times in the last year. The patient had a history of anxiety and was on lamotrigine (150 mg PO daily) and sertraline (200 mg PO daily).

At admission, the patient presented with mild hypokalemia (3.3 mmol/L) and hyperchloremia (108 mmol/L); otherwise, labs were unremarkable. Vital signs were stable. UDS was positive for cannabinoids. The patient reported a history of marijuana use intermittently since 10th grade and then regularly since 11th grade (the patient is now in university). The patient also reports taking edibles (10 mg) on occasion and vapes nicotine (about 1 pen/week).

At the outside hospital, the patient received IV fluids, prochlorperazine, diphenhydramine, and lorazepam before transferring. On arrival, her EKG showed a slightly prolonged QTc of 474 ms. Patient B was admitted to our hospital for dehydration and CHS management. Given the prolonged QTc, the patient was trialed on lorazepam, diphenhydramine, and ondansetron IV as needed. The patient continued with multiple episodes of emesis overnight despite three doses of IV lorazepam and one dose of IV diphenhydramine. Per the nurse, diphenhydramine seemed to worsen her symptoms. The EKG showed her QTc was still 468 ms. After discussion of the new QTc results, the care team decided to start haloperidol 1 mg intramuscularly (IM) x1 and capsaicin topical 0.025% cream three times daily (TID), while continuing lorazepam 2 mg IV q6hr as needed (PRN). Overnight, the patient received two doses of IV lorazepam and one dose of IM haloperidol with six episodes of emesis. Despite some initial improvement, the patient ultimately reported that haloperidol did not fully help, and on day 3, the physician started NK-1 antagonists, as a series of 3 doses starting with fosaprepitant 115 mg IV. The patient’s nausea and vomiting significantly improved. On day 5, her QTc was unchanged (461 ms). Patient B had two episodes of emesis overnight and received one dose of IV lorazepam 2 mg. On day 6, the patient was still improving without any emesis but still with retching. She received three doses of fosaprepitant and was started on a scopolamine patch in preparation for the flight home. Patient B was discharged home on day 7 with capsaicin topical 0.025% cream TID, ondansetron 8 mg PO Q8h PRN, and lorazepam 2 mg PO Q6h PRN. Upon discharge, the family reported that they plan to seek out further care for potential marijuana/nicotine cessation.

## 3. Discussion

Cannabinoid Hyperemesis Syndrome (CHS) becomes more prevalent with increasing cannabis use and is commonly resistant to traditional antiemetics [13,14]. Limited dedicated guidelines exist for early diagnosis and treatment, especially in pediatric patients. Current management is primarily based on evidence collected from case series, reports, and small retrospective clinical studies. A recent study demonstrated that non-traditional antiemetics (such as benzodiazepines, butyrophenones, and topical capsaicin) appear more effective in resolving patient symptoms than traditional antiemetics (such as serotonin (5-HT_3_) antagonists and phenothiazines, antihistamines, metoclopramide, or trimethobenzamide) [15]. The most reported antiemetics effective for CHS include benzodiazepines, haloperidol, and capsaicin cream.

A recent randomized controlled trial compared the efficacy of IV haloperidol (0.05 mg/kg or 0.1 mg/kg) with IV ondansetron (8 mg) for CHS management [17]. The study showed that haloperidol at either dose was superior to ondansetron for acute treatment of CHS in the ED (*p* < 0.05), with similar improvements in both pain and nausea, less use of rescue antiemetics (31% versus 59%), and shorter duration of emergency room stay (3.1 h versus 5.6 h, *p* < 0.05). Haloperidol has shown effectiveness when administered both intravenously and intramuscularly, but IV administration appears to be associated with a higher risk of QTc prolongation and TdP [18]. As of now, no head-to-head comparison studies have been conducted in patients, and the optimal haloperidol dosing regimen for the acute management of CHS has yet to be determined [13].

A common concern about using haloperidol for CHS treatment is its potential to induce QT prolongation. QT prolongation is the most common cardiac arrhythmia among teenage cannabis users, with an incidence rate of 513.1 per 100,000 cases, followed by palpitations at 139.5 and atrial fibrillation at 116.3 per 100,000 cases [7]. The prevalence of QTc prolongation in teenage cannabis users is relatively rare but is severe and life-threatening if it occurs [10,11,12,19,20]. The development of QTc and TdP is thought to result from multiple factors, collectively, including female gender; consumption of high doses and/or prolonged use of cannabis products; previous medical history of QTc or TdP; significant family history of QTc, TdP, or cardiac arrest; underlying medical conditions predisposing QTc (e.g., electrolyte abnormalities, left ventricular failure, Brugada syndrome); and concurrent use of other QTc medications [10,12,19,21,22].

As summarized in Table 1, severe cardiovascular adverse events, such as QTc prolongation and TdP, have been reported among adolescents and young adult cannabis users during the management of CHS. Consistent with our two patient cases, previously reported subjects were female and between 18 and 22 years of age. Hypokalemia was observed in three of five reported cases and in our two patients. An earlier retrospective study showed that potassium below 3.0 mmol/L predicted QT prolongation >500 msec among CHS patients [23]. In addition, one patient received haloperidol for nausea. Two cases were found retrospectively to have arrhythmogenic genetic mutations, one of which was complicated by hypokalemia and administration of QT-prolongation medications [19,20].

In our cases, there were no previous records of an EKG exam in their medical histories. For patient A, EKG monitoring was only performed after administering additional QTc-prolonging medications (haloperidol and ondansetron) and when the patient was experiencing tachycardia and chest pain, which identified QTc prolongation. Aware of the risk of QTc prolongation, the clinical team requested an EKG on the second patient upon admission, noting a borderline QTc interval. The patient was given a trial dose of haloperidol with close monitoring. However, due to concerns about QTc prolongation, the haloperidol dose was limited to 1 mg (0.015 mg/kg). The lack of efficacy could be attributed to the low dose of haloperidol. In a recent case series report of acute treatment of CHS in adolescents, patients received 5 mg IV haloperidol combined with lorazepam and/or capsaicin for complete acute symptom relief [24].

Droperidol has a mechanism of action similar to that of haloperidol, belonging to the butyrophenone family. In the ED setting, droperidol has been shown to produce more rapid sedation [25]; however, a recent study (2014) failed to identify any significant differences between these two medications in adverse events and QTc prolongation when used by paramedics [26]. There is limited efficacy evidence available using droperidol for CHS acute management. A retrospective study of CHS cases showed that the droperidol treatment reduced the median length of stay compared to other antiemesis treatments (e.g., ondansetron and metoclopramide), and the most used dose was 0.625 mg IV [27]. Although Children’s Minnesota opted to use droperidol for acute CHS management due to its “lower risk of potential QTc prolongation and extrapyramidal side effects,” the choice between droperidol and haloperidol may vary by institutions based on their hospital formulary, as there is a lack of comparative safety and efficacy studies between the two [28].

Based on the current clinical evidence, we recommend the following procedures to minimize the risk of QTc prolongation when using haloperidol for CHS acute management.

**(1) Conduct a thorough patient assessment,** including cannabis usage, social history, family history, medication history, and medication reconciliation, preferably taken in the absence of parents or other involved adults and corroborated by urine drug screening. The presentation of CHS indicates chronic use of cannabis products, which tends to occur in people who use cannabis at least once a week (97.4%) [29]. A study showed that two-thirds of patients with CHS use cannabis at least once daily [30]. CHS is also observed more often in adults who have been using cannabis since their adolescent years. There may be a delay of up to 19 years in the onset of CHS symptoms preceded by chronic marijuana misuse in nearly all cases [31,32].

**(2) Order initial labs,** including a complete metabolic panel and serum magnesium levels, to rule out electrolyte abnormalities. Hypocalcemia, hypokalemia, and hypomagnesemia are known risk factors for prolonged QTc and TdP. Frequent and prolonged vomiting may cause electrolyte abnormalities, such as hypokalemia. If the patient has abnormal electrolyte levels, immediate correction is essential.

**(3) Monitor QTc interval with haloperidol antiemetic treatment in patients presenting risk factors**. Based on an earlier study of 282 cannabis users admitted for CHS, haloperidol treatment did not significantly increase the QTc measured by EKG [23]. However, EKG monitoring is highly recommended for patients with significant risk factors, i.e., a history of cardiovascular disease, for which a baseline EKG is necessary. A clinical CHS management guideline developed by Children’s Minnesota suggests QTc prolongation thresholds of >460 msec for males and >480 msec for females. A cardiologist should be consulted if QTc > 500 msec even after correcting electrolyte imbalances [28].

A case report showed that haloperidol led to agitation and worsening of symptoms in a 21-year-old female patient [33]. If the patient does not respond to haloperidol treatment or has significant risk factors of QTc prolongation, the following antiemetic medications can be considered for the acute management of CHS without posing a significant risk of QTc prolongation.

- Lorazepam (Benzodiazepine): The possible mechanism of action might be related to enhancing the inhibitory effect of gamma-aminobutyric acid (GABA) on neuronal excitability, which results in decreasing the activation of cannabinoid receptor CB1. Reinert’s (2021) systematic review of pediatric patients concluded that benzodiazepines were the most reported efficacious agents for CHS acute management, followed by topical capsaicin and haloperidol in pediatric patients [14]. Due to the potential for respiratory distress, if a patient’s UDS indicates concurrent opioid use, benzodiazepines for acute CHS management should be used cautiously and under close monitoring.
- Topical capsaicin (0.025% and 0.075% cream): Capsaicin is a neuropeptide-active agent that affects transient receptor potential vanilloid-1 (TRPV1) receptors, leading to the sensation of heat, which can relieve nausea associated with CHS. It should be applied topically to the abdomen, back, chest, or back of the arms.

Mixed adult and adolescent retrospective studies have been reported using the 0.025% capsaicin cream. The first case series was reported in 2017, using capsaicin for CHS management in adolescents [34]. Two adolescents with CHS who had previously failed to respond to standard antiemetic therapies showed remarkable improvement with 0.025% capsaicin cream treatment; their symptoms either resolved or significantly improved within just 30 min.

A retrospective study involving both adults and adolescents examined the use of 0.025% capsaicin cream for CHS management [30]. This study revealed promising outcomes, with 55% of patients requiring no more than one additional medication for symptom relief. Patients treated with capsaicin experienced a shorter time to discharge following treatment. The presence of nausea was identified as a significant predictor of the cream’s efficacy. However, subgroup analysis suggested capsaicin cream might be less effective in patients under 21. In addition, patients with a longer duration of symptoms (≥1 month) were less likely to experience symptom relief with capsaicin. The study concluded that capsaicin may be considered early in the ED management course or as a part of a multi-model approach in combination with other antiemetics. Another adult study also showed that patients who received capsaicin earlier within their first two rounds of treatment had a significantly shorter length of stay than patients who received capsaicin later [35].

A recent randomized, placebo-controlled pilot study in adults (mean age 32 years) demonstrated that 0.1% capsaicin significantly improved nausea at 60 min, and a higher proportion of patients (29.4%) achieved complete nausea relief [36].

Of note, adverse events, including painful burning sensations, were also observed in previous studies requiring the removal of the cream [35,36]. For pediatric patients, it is recommended to use a small amount and watch for any adverse reactions (e.g., blistering of skin, irritation).

- Aprepitant and fosaprepitant (Neurokinin-1/NK receptor antagonist): NK-1 antagonists were first developed for chemotherapy-induced nausea and vomiting and exhibit promising antiemetic efficacy by blocking the action of substance P at the NK-1 receptor in the brain and CNS [37,38]. Recent studies examining the safety of antiemetic regimens containing NK-1 antagonists found that the drug has few adverse cardiac effects [39,40].

Using NK-1 antagonists (aprepitant/fosaprepitant) for cyclic vomiting syndrome (CVS) has been reported in recent years. An earlier study conducted in 2014 reported that 41 pediatric patients with CVS who had failed or discontinued previous medical treatment due to severe side effects showed an 81% response to prophylactic aprepitant and a 76% response to abortive treatment [41]. Another case report (2021) involving a 13-year-old girl with severe CVS demonstrated that a 3-day course of aprepitant (125 mg on day 1 and 85 mg on days 2–3) administered approximately every two weeks effectively prevented new vomiting cycles at home, with no further episodes occurring since the initiation of aprepitant [42]. The most recent retrospective study reviewed 70 adult patients with CVS who were prescribed a 3-day course of aprepitant [43]. Most patients (71%) had a global response to the treatment with significantly reduced ED visits and number of CVS episodes.

However, data on using NK-1 antagonists for CHS management are very limited and have mainly been reported in adults [44]. A recently published abstract reported that 30 pediatric patients with CHS (ages 15–18, 77% females) all received at least one dose of aprepitant [45]. The majority (77%) were prescribed a 3-day course, with the duration of treatment ranging from a single dose to a 5-day course. Ninety-seven percent of patients (29/30) experienced overall improvement. Adolescent patients tolerated aprepitant well, and no side effects were reported. In our third patient case, significant symptom relief was achieved with a three-dose course of aprepitant.

- Olanzapine (antagonist of dopamine D2 receptors and serotonin 5HT2A receptors): Olanzapine is a second-generation psychotropic for schizophrenia and bipolar disorder. Since the 2000s, olanzapine has been increasingly used in the management of chemotherapy-induced nausea and vomiting as recommended by the American Society of Clinical Oncology (ASCO) and the National Comprehensive Cancer Network (NCCN) [37,38,46]. Olanzapine rarely causes QTc prolongation and has also been reported for use in the acute management of CHS [28]. A case series reported using olanzapine for treatment-refractory CHS, which is effective in symptomatic treatment, especially when patients present with comorbid psychotic symptoms or agitation [47]. However, the efficacy and effective dosing still need to be defined [48,49].

**(4) Develop an algorithm for managing QTc prolongation and TdP in the ED and provide regular education and training to the multidisciplinary team.** Pharmacists play an essential role in managing QTc prolongation and TdP when it occurs. Drugs that cause QTc prolongation should be identified and discontinued immediately. Treatment includes intravenous magnesium sulfate and potentially potassium chloride and/or calcium gluconate if these electrolytes need to be repleted. In refractory cases of recurrent TdP that is bradycardia-mediated (not due to congenital long QT syndrome), isoproterenol may be considered for off-label use as a continuous infusion, titrated to clinical response. If necessary, defibrillation or cardioversion may be performed.

One limitation of the analysis of our two patient cases is the lack of detailed information about their cannabis use history, such as duration, methods of consumption, frequency, dose, and any other potentially relevant details, which are crucial for better understanding the potential association between cannabis use and CHS, as well as improving diagnosis. However, such information was not recorded in patient medical records, representing a notable limitation of this retrospective case report. Previous studies have also reported that positive substance use histories are rarely documented and that there is often poor concordance between documented and self-reported substance use [50]. A prospective study ensuring confidentiality with a waiver of parental consent, validated with substance use screening, could help overcome potential barriers and improve medical record documentation. Future comparative studies are warranted to identify effective antiemetic regimens for the management of CHS.

## 4. Conclusions

Cannabinoid Hyperemesis Syndrome (CHS) has been increasingly seen in adolescents and young adults with chronic cannabis use. CHS patients are at an increased risk of QTc prolongation due to prolonged cannabis use, electrolyte imbalances commonly associated with hyperemesis episodes, medications, and other risk factors. Our cases, along with those cited in the literature, underscore the importance of monitoring QTc in CHS patients admitted for acute antiemetic management, particularly when initiating haloperidol treatment. If a patient presents with risk factors for QTc prolongation, non-haloperidol antiemetic drugs such as topical capsaicin, lorazepam, aprepitant/fosaprepitant, and olanzapine are preferred for acute management.

## Figures and Tables

**Table 1:** Previously published cases of QTc prolongation and/or TdP in frequent adolescents and young adult users of cannabis.

Author	Design	Patient(s) Demographics	Cannabis Use	Clinical Outcomes
Patel et al. (2021) [12]	Case report	A 21-year-old female without a history of congenital prolonged QT, with QTc interval WNL (430 msec) on prior admissions and minimal medical history.	Daily heavy cannabis habit with vomiting spells over the prior 2–3 months	Intractable vomiting, hypokalemia (2 mEq/L), and QTc prolongation (603 msec) led to seizures and TdP, which resulted in ventricular fibrillation with no pulse; ROSC attained after 1 round CPR; was extubated the following day and confessed to daily heavy cannabis habit; educated on strict cessation of marijuana for prevention.
Shah et al. (2021) [10]	Case report	A 19-year-old female with a family history of prolonged QTc (mother/sister) and family history of prolonged QTc followed by sudden cardiac arrest (maternal cousin/maternal aunt) but a personal prior history of QTc WNL (454 msec).	Frequent use of marijuana through wax vaping (dabbing)	Witnessed episode of loss of consciousness for which family performed CPR for 20 min; defibrillated for the initial rhythm of V.F. and achieved ROSC; EKG showed QTc prolongation (622 msec); two more episodes of TdP after admission; genetic testing identified a genetic mutation for long QT syndrome type 2 (LQTS2); received an implantable cardioverter–defibrillator and started on beta-blockers.
Von Both et al. (2021) [19]	Case report	A 22-year-old female with anxiety and a 3.5-year history of cyclic vomiting, with recent hypokalemia, borderline prolonged QT interval (462 msec), and receipt of several QT-prolonging medications. She was found postmortem to have undiagnosed genetic variants associated with arrhythmias and cardiomyopathy.	Cannabis use since age 14 except for an ineffective 3-month abstinence trial	Two cardiac arrests were experienced during a “bounce-back” ED visit for nausea and vomiting, during which she presented with bradycardia, hypertension, dehydration, and hypokalemia; she achieved sustained ROSC but remained in a vegetative state due to post-cardiac arrest hypoxic-ischemic brain injury until neurological declaration of death pronounced several days later. Death was attributed to a fatal cardiac arrhythmia complicating vomiting-induced hypokalemia and treatment with QT-prolonging and potentially arrhythmogenic medications, with the cardiac genetic mutations contributing.
Kwag et al. (2022) [20]	Case report	A 20-year-old female with endometriosis, pelvic inflammatory disease, and a recent ED visit for epigastric pain, diarrhea, nausea, and vomiting, for which haloperidol was administered.	Frequent cannabis use, three to four times per week	Chest pain and shortness of breath led to “bounce-back” admission with prolonged QTc of 477 ms, during which haloperidol, ondansetron, and lorazepam were given for nausea; syncope occurred with subsequent nausea, for which haloperidol was given; recurrent chest pain with prominent U waves and persistently prolonged QTc (489 msec); U waves persisted beyond discharge.
		An 18-year-old female with a medical history of Premature Ventricular Contractions (PVC), Premature Atrial Contractions (PAC), and persistent tachycardia.	Recent consumption of cannabis-containing brownies and urine positive for cannabinoids	Tinnitus, tachycardia, lightheadedness, hypokalemia (3.3 mmol/L), and vomiting, for which ondansetron was given; prolonged QTc (566 msec); tachycardia that resolved after potassium repletion, correction of potassium level, and normalization of QTc to 413 msec; discharged.

CPR, cardiopulmonary resuscitation; EKG, electrocardiogram; EMS, emergency medical services; ICD, implantable cardioverter–defibrillator; ICD-10-CM, International Classification of Diseases, Tenth Revision, Clinical Modification; LVEF, left ventricular ejection fraction; ROSC, return of spontaneous circulation; TdP, Torsades de Pointes; VF, ventricular fibrillation; VT, ventricular tachycardia; WNL, within normal limits.

## Data Availability

Data are contained within the article.
